# Gestational Trophoblastic Disease with Coexisting Progressing Pregnancy: Personalised Treatment Modalities

**DOI:** 10.1155/2023/5502317

**Published:** 2023-10-26

**Authors:** Elena Ulrikh, Elena Dikareva, Igor Govorov, Eduard Komlichenko, Tatiana Pervunina, Olga Li, Oksana Zhamborova, Aminat Dzharbaeva, Viktor Deynega, Veronika Artemenko, Adel Urmancheeva

**Affiliations:** Personalized Medicine Center, Almazov National Medical Research Centre, Saint-Petersburg 197341, Russia

## Abstract

**Purpose:**

Gestational trophoblastic disease (GTD) coexisting with a steadily progressing pregnancy is an extremely rare condition presented in the literature as a single case or case series of successful delivery. The purpose of this study was to describe five cases of GTD and present possible management strategies for such patients.

**Methods:**

Clinical data of five pregnancies with coexisting GTD were identified within the Almazov National Medical Research Centre from 2018 to 2021.

**Results:**

Three cases of multiple pregnancies with complete hydatidiform moles and two cases of singleton pregnancies with intraplacental choriocarcinoma and invasive hydatidiform moles were identified. Three pregnancies were prolonged and ended with preterm deliveries. Malignant transformation of the GTD accounted for 60% of the cases. The condition of newborns was based on the level of prematurity and functional immaturity, and in all cases, it was aggravated by anemia.

**Conclusion:**

GTD coexisting with progressing pregnancy is threatened by the risks of preterm delivery, miscarriage, hemorrhage, and disease progression and requires monitoring in a multidisciplinary clinic experienced in the management of patients with malignant tumors during pregnancy. In cases of prolonged pregnancy against the background of GTD, we suggest the following monitoring during pregnancy: pelvic, abdominal ultrasound/MRI (without contrast), prenatal invasive fetal karyotype testing in cases of singleton pregnancy, lung X-ray/CT with uterine shielding, weekly assessment of *β*-hCG levels, and dynamic monitoring of the fetus. The following postnatal monitoring should be performed: morphological examination of the placenta, weekly assessment of *β*-hCG levels up to normalization, then monthly assessment up to six months, and control of *β*-hCG level of the newborn.

## 1. Introduction

Gestational trophoblastic disease (GTD) is a rare condition that involves a spectrum of proliferative trophoblast disorders associated with pregnancy. In European countries, GTD is diagnosed in 0.6–1.1 cases per 1,000 pregnancies, and data from population registries suggest age, geographic, and ethnic differences in the incidence of trophoblastic tumors [1, 2]. GTD is the result of an aberrant human pregnancy with an abnormal karyotype due to defect in gametogenesis or fertilisation and abnormal trophoblastic proliferation [3].

In some cases, GTD can coexist with a normal fetus, which is more often observed in multiple pregnancies [4, 5]. Such pregnancies (usually twin pregnancies) combine a developing fetus and a molar pregnancy (complete or partial moles) and occur in one case per 22,000 pregnancies to one case per 100,000 pregnancies [6, 7]. Gestational trophoblastic neoplasia (GTN), such as intraplacental choriocarcinoma, detected against the background of a developing pregnancy, is an even rarer situation in obstetrics [8–10]. In the UK, choriocarcinoma develops in around one in 50000 deliveries [3]. Prenatal diagnosis of GTN in these situations is difficult and requires careful weighing of the risks of pregnancy prolongation/termination. The literature data concerning prolongation of such pregnancies are controversial, showing the variability of successful live births altering from 13 to 63% [1, 2]. There are still difficulties in choosing strategies for monitoring or treatment of the disease during coexisting pregnancy [11–13]. We present a report of five clinical cases of GTD, the management tactics for these patients, and a review of the literature on this topic.

## 2. Methods

All cases of GTDs against the background of developing pregnancy treated at the Almazov National Medical Research Centre, with different histological forms and clinical outcomes, were analyzed for the period from 2018 to 2021. The average follow-up period after treatment was 18.3 months (range: 7–29 months).

Determination of *β*-human chorionic gonadotropin (*β*-hCG) serum levels was performed to diagnose GTD, followed by weekly *β*-hCG measurements to monitor the course of the disease. To clarify the extent of the pathological process, all patients underwent pelvic and abdominal ultrasound/MRI (1.5T scanner) and chest radiography with uterine shielding.

Morphological verification of the GTD was performed in all cases by histopathological examination of placenta after delivery or products of conception after curettage/vacuum aspiration. Immunohistochemical markers such as Ki-67, proliferating cell nuclear antigen (PCNA), p53, and p57kip2 were determined in the morphological material for differential purposes. Prenatal fetal karyotype analysis was performed in case of prolonged singleton pregnancy to exclude chromosomal abnormalities.

Since the primary objective was to describe five cases of GTD and to present possible management tactics for patients with GTD and coexisting progressing pregnancy, we mainly utilized descriptive statistics.

## 3. Results

Through 2018–2021 period, five cases of GTD with coexisting developing pregnancies were identified in the Almazov National Medical Research Centre: two cases of multiple pregnancies with complete and partial hydatidiform mole, one case of multiple pregnancy with choriocarcinoma, and two cases of singleton pregnancies - one with choriocarcinoma and the other with invasive hydatidiform mole.

The mean age of pregnant women at the time of GTD was 33.2 years. The patient characteristics and anamnestic data are presented in Table 1.

Ultrasound combined with determination of *β*-hCG levels, which exceeded the reference values for the given gestational age, was the main method for diagnosis of GTD (shown in Table 1). Pelvic ultrasound revealed features of the disease—the presence of small cystic changes, increased echogenicity in the structure of the placenta, myometrium, or decidua, and picture of a “snowstorm.” In one patient (N5), bilateral theca-lutein ovarian cysts were diagnosed. In three cases (N1, N2, and N5), GTD was diagnosed in the first trimester of pregnancy (before 12 weeks) and in two patients (N3 and N4) in the second trimester (16/17 weeks and 19/20 weeks). In four cases, pregnancies occurred spontaneously, and in one case (N4) after the third attempt of *in vitro* fertilisation (IVF) technology.

In all cases, the *β*-hCG level at the time of diagnosis was significantly higher than the reference value for GW (>285000 Ul/L). In two cases (N1 and N2), the *β*-hCG level was higher than 400000 Ul/L, decreasing to 200000 Ul/L in one case (N1) and staying above 550000 Ul/L throughout the pregnancy in the other case (N2). In one instance (N4), GTD was identified at 19/20 GW with a heightened *β*-hCG serum level (285384 Ul/L), reaching a peak of 402000 Ul/L throughout pregnancy. Patient N5 had the highest *β*-hCG level at the time of GTD detection, which was 709300 IU/L at 11 GW, and it increased to 732486 Ul/L over the observation period. Consequently, the serum *β*-hCG levels were mostly above the reference values throughout the observation period. The only exception was patient N3 with choriocarcinoma, whose *β*-hCG level was above the reference value (470541 IU/L) when GTD was detected at 16/17 GW, but then dropped to 80827 IU/L by 22 GW and thereafter was in the range of 60000–80000 IU/L throughout the pregnancy.

In all cases, pregnancies were complicated by the threat of miscarriage, metrorrhagia, pain, and anemia. Ultrasound and MRI were used to diagnose four women (N1, N2, N3, and N5) with subchorionic/subamniotic hematomas. The results are presented in Table 1.

According to a clinical investigation during pregnancy (pelvic and abdominal ultrasound/MRI and chest radiography with uterine shielding), no evidence of distant metastases was found. In case of singleton pregnancy (N3), in which the woman decided to prolong the pregnancy with GTD, amniocentesis combined with karyotype analysis of the developing fetus was performed in the second trimester. Chromosomal abnormalities were not detected.

In three cases (N2, N4, and N5), GTD was diagnosed against the background of a developing multiple pregnancy (diamniotic dichorionic twins), with pathological lesions in one amnion in N2 and N4 cases and in both twins in the N5 case.

In all cases of advanced pregnancy with GTD, a multidisciplinary team was responsible for determining the subsequent treatment course. The decision to extend pregnancy is contingent upon a few conditions: no signs of cancer, no fetal deformities, a normal karyotype, and moderate obstetric risks. Pregnant women were informed of the potential complications of pregnancy, risk of malignant transformation, and possible effects on the fetus. A woman's agreement to continue her pregnancy was legally recorded.

### 3.1. Pregnancy Outcomes

Pregnancy termination was performed in two cases (N1 and N5): in one case of diamniotic dichorionic twin with partial hydatidiform mole of both fetuses (N5) due to heavy bleeding and high risk of adverse obstetric outcomes, and in one case of single pregnancy with invasive hydatidiform mole (N1) due to progressive retrochorial hematoma, suspected trophoblastic tumor invasion into the myometrium according to ultrasound/MRI, and high risk of adverse maternal outcomes.

In two cases (N3 and N4), the multidisciplinary team, after assessing the diagnostic results, cancer risk, risk of complications during pregnancy, absence of fetal abnormalities, and women's desire to continue the pregnancy, concluded that pregnancy could be safely extended. In one instance (N2), the patient refused to terminate the pregnancy despite MRI evidence of trophoblastic tumor invasion into the myometrium, which posed a high risk of malignant transformation and obstetric risk. In all cases, close observation of the patient and the fetus was conducted (fetal monitoring, *β*-hCG control, ultrasonography, and MRI). All three cases experienced pregnancy complications, including threatened miscarriage, bleeding, pain syndrome, anemia, and thyrotoxicosis, and necessitated medical intervention.

All prolonged pregnancies in women with GTD were realized in preterm deliveries at 27 0/7 weeks (N2), 28 0/7 weeks (N3), and 33 4/7 weeks (N4). In two cases (N3 and N4), preterm spontaneous labor ended in natural childbirth, and in one case (N2), emergency caesarean section and hysterectomy with fallopian tubes were performed against the background of life-threatening hemorrhage.

The condition of the newborns was consistent with their gestational term; all exhibited signs of prematurity and functional immaturity, and the intensity of these signs varied depending on the term of childbirth. Two newborns (N2 and N3) were diagnosed with severe anemia that required blood transfusion. No deformities or tumors were observed in any of the newborns.

### 3.2. GTD Outcomes

Postpartum or postevacuation morphological investigation revealed choriocarcinoma in two cases (N2 and N3) and molar pregnancies in three cases (N1, N4, and N5). GTN accounted for 60% of the cases and included invasive molar pregnancy (N1) and intraplacental choriocarcinoma (N2, N3). The diagnosis of choriocarcinoma was made after immunohistochemical examination of placental tissue and determination of Ki-67, PCNA, p53, and p57kip2. Both patients with choriocarcinoma (N2 and N3) showed signs of retroplacental vascularization/neovascularization on pelvic MRI, which are considered by some authors as signs of an invasive pathological process. However, the small number of observations required further study. Remarkably, newborns from mothers with GTN were diagnosed with severe anemia at birth, requiring hemotransfusion in 100% of the cases (N2 and N3).

After pregnancy, regardless of the outcome, a complex of diagnostic procedures was performed to detect pathological changes in the pelvic organs and to rule out metastatic lesions of the thorax, abdomen, and brain. No pathologic changes in the pelvic organs (uterus, ovaries, and vagina) were detected. In the postnatal period, the *β*-hCG titer was monitored (Table 1). The data allowed us to assess the presence of risk factors for the disease and to choose the appropriate tactics for each case.

In three cases (N1, N2, and N3), standard chemotherapy with methotrexate/folinic acid (Mtx/FA) was required, taking into account the sum of the FIGO-WHO scores and determination of the resistance risk group. Complete regression of the tumor during first-line chemotherapy was observed in two patients (N2 and N3) with GTD. One patient (N1), due to drug resistance (after first-line Mtx/FA), received second-line chemotherapy (dactinomycin 500 mcg from day 1 to day 5, with a complete response). All patients (*n* = 5) were in remission during follow-up.

## 4. Discussion

It is uncommon to have a combination of GTD and developing pregnancy, including multiple pregnancies. Extending the pregnancy in this case always carries a high risk of various complications for both the mother and unborn child. A potential shortcoming of this study is the limited sample size (due to the infrequency of GTD). Retrospective studies published in the past two decades (Table 2) suggest that prolongation of pregnancy is achievable when gestational trophoblastic disease is detected, with live birth rates ranging from 13 to 63% and babies being born at an average of 30 weeks of gestation [4, 5, 14–18]. The rate of maternal complications, including hemorrhage, hyperthyroidism, and preeclampsia, is reported to be approximately 80%, and up to 34% of the total cases are eventually identified as trophoblastic disease [8, 17, 19]. It is essential to diagnose GTN early and assess the risks of early GTN in pregnancy that is still developing. As the number of observations is limited, there is currently no definitive criterion for identifying a GTN [4, 20, 21]. For many years, authors have been divided on the issue of the risks of GTD malignancy in pregnant women, but in recent decades, it has been demonstrated that continuing pregnancy does not increase the risk of GTN [4, 9].

**Figure 1:**
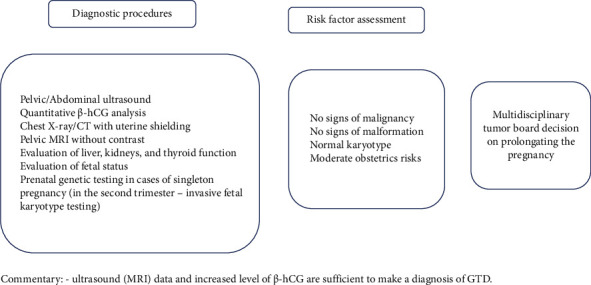
Diagnostic algorithm for patients with GTD and coexisting progressing pregnancy.

**Figure 2:**
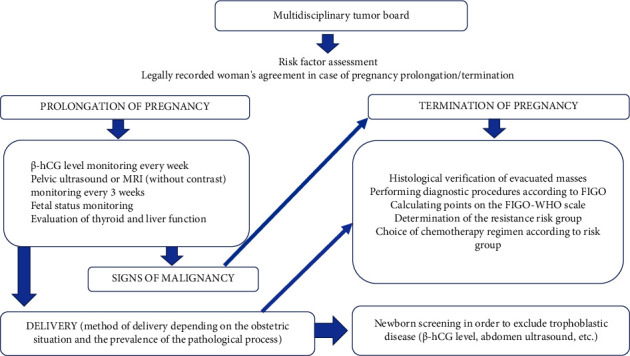
Treatment algorithm for patients with GTD and coexisting progressing pregnancy.

Drawing on the literature review and case series presented, we created algorithms for the management of GTD in developing pregnancy (Figures 1 and 2). Therefore, in cases of prolonged pregnancy accompanied by GTD, it is essential to perform the following steps:

1. Pelvic, abdominal ultrasound/MRI without contrast
2. Prenatal invasive fetal karyotype testing in cases of singleton pregnancy
3. Lung X-ray/CT with uterine shielding
4. Weekly assessment of *β*-hCG levels
5. Dynamic monitoring of the patient and the fetus in a multidisciplinary perinatal center with experience in managing such patients
6. Morphological examination of the placenta after delivery
7. Postnatal monitoring (pelvic ultrasound/MRI with contrast; chest, abdominal ultrasound/CT; MRI of the brain in case of pulmonary metastases; *β*-hCG assessment weekly up to normalization (not allowing plateau in four consecutive samples during three weeks), then monthly up to six months)

## 5. Conclusion

GTD coexisting with progressing pregnancy is threatened by the risks of preterm delivery, miscarriage, hemorrhage, and disease progression and requires monitoring in a multidisciplinary clinic experienced in the management of patients with malignant tumors during pregnancy.

## Figures and Tables

**Table 1:** Description of pregnant women with GTD and management and outcomes of GTD after pregnancy.

Case	Age (y)	Obstetric anamnesis	Time of GTD detection (GW)	P type	P outcome	Ultrasound data	MRI data	Histological form	*β*-hCG titer (3 weeks postnatal follow-up)	Presence of metastatic lesions (localization)	Treatment
N1	31	P-0	11	Singleton pregnancy	Termination of pregnancy for obstetrical/medical reasons	Hyperechogenic reticular, cellular structure. Retrochorial hematoma.	Multicystic pathological formation, numerous vessels. The contact area is uneven, indistinct. Retroplacental vascularization/neovascularization. Adhesion areas (5.0–5.5 cm). Subchorionic hematoma. Mild collateralization.	Invasive mole	Plateau	Pulmonary	Chemotherapy Mtx/FA, dactinomycin

N2	36	P-4, A-1, D-3, Ln-3	10	Diamniotic dichorionic twins	Childbirth 27 GW	Hyperechogenic formation of heterogeneous structure with anechogenic multifaceted inclusions. Retrochorial hematoma.	The lower amniotic cavity and placenta are replaced by pathological tissue of heterogeneous cystic structure. Contours indistinct, rough, infiltration of the anterior uterine wall. Collateralization in the inferior segment. Retroplacental vascularization/neovascularization. Subamniotic hematoma.	Choriocarcinoma in one placenta	Plateau	Pulmonary	Chemotherapy Mtx/FA

N3	32	P-2, A-1, D-1, Ln-1	16	Singleton pregnancy	Childbirth 28 GW	One-third of the placenta with cystic inclusions. Subamniotic hematoma.	Cellular structures of irregular shape. The surface of the placenta is rough, with a lumpy contour. Retroplacental and intramural vascularization.	Intraplacental choriocarcinoma	Plateau	Pulmonary	Chemotherapy Mtx/FA

N4	36	P-0	19	Diamniotic dichorionic twins	Childbirth 33 GW	The formation of a cellular structure.	Placental overgrowth in the form of cysts of various diameters.	Noninvasive mole of a single fetus	Normalisation	Absent	None, observation

N5	31	P-1, D-1, Fd-1	11	Diamniotic dichorionic twins	Termination of pregnancy for medical reasons	Formation of a cellular structure. Theca lutein cysts. Subamniotic hematoma.	Multicystic masses surround the amniotic cavity, subtotally filling the uterine cavity. Majority parts of both placentas are represented by pathological multicystic structures. Subamniotic hematoma. The boundaries with myometrium are clear, smooth. Theca lutein cysts.	Noninvasive mole of both fetuses	Normalisation	Absent	None, observation

P, pregnancy; D, deliveries; A, abortion; Ln, live newborns; Fd, fetal death; GW, gestational weeks; Mtx, methotrexate; FA, folinic acid.

**Table 2:** Pregnancy and GTD outcomes (literature review and own experience).

Authors	Country	Number of cases	Abortion	Termination of pregnancy for obstetric reasons	Miscarriage or fetal death	Childbirth	Trophoblastic neoplasia
Fishman et al. [4]	USA	7	0	5 (71%)	0	2 (29%)	4 (57%)
Wee and Jauniaux [14]	UK	8	1 (12.5%)	0	2 (25%)	5 (62.5%)	3 (37.5%)
Niemann et al. [5]	Denmark	8	5 (62.5%)	0	2 (25%)	1 (12.5%)	2 (25%)
Lee et al. [15]	Korea	6	1 (17%)	2 (33%)	2 (33%)	1 (17%)	3 (50%)
Kihara et al. [17]	Japan	7	2 (29%)	0	4 (57%)	1 (14%)	1 (14%)
Kutuk et al. [16]	Turkey	5	2 (40%)	0	2 (40%)	1 (20%)	1 (20%)
Hemida et al. [18]	Egypt	12	5 (41.7%)	2 (16.7%)	5 (41.7%)	3 (25%)	1 (8.3%)
The current study	Russia	5	0	2 (40%)	0	3 (60%)	3 (60%)
Total		58	16 (27.6%)	11 (19%)	17 (29.3%)	17 (29.3%)	18 (31%)

## Data Availability

The data used to support the findings of this study are included within the article.
